# Editorial: Co-creating future social services

**DOI:** 10.3389/fsoc.2025.1619854

**Published:** 2025-06-03

**Authors:** Andrzej Klimczuk, Robin Miller, Carola Orrego, Hilla Dolev

**Affiliations:** ^1^Department of Social Policy, Collegium of Socio-Economics, SGH Warsaw School of Economics, Warsaw, Poland; ^2^Department of Social Work and Social Care, School of Social Policy, University of Birmingham, Birmingham, United Kingdom; ^3^Avedis Donabedian Foundation, Barcelona, Spain; ^4^Avedis Donabedian Research Institute, Universitat Autònoma de Barcelona, Barcelona, Spain; ^5^Regulation and Quality Assurance Team, Myers-JDC-Brookdale Institute, Jerusalem, Israel; ^6^Paul Baerwald School of Social Work and Social Welfare, The Hebrew University of Jerusalem, Jerusalem, Israel

**Keywords:** co-creation, social inclusion, social innovation, social policy, social services, social work, welfare state

## Overview

This Research Topic is an extension of the 32nd European Social Services Conference (Antwerp, Belgium, 26–28 June 2024), the European Social Network's annual event. This edition focused on co-creating future community-based social services. Event participants and others working or researching in this field were invited to submit their theoretical and empirical contributions examining co-creation regarding urban social inclusion, workforce management, and digital social service solutions. Emphasis was placed on challenging and refining the sociological, social policy, and social work theories that underpin assumptions about co-production, personalization, social inclusion, and diversity in service provision and evaluation.

Four journals were involved in this project: “Frontiers in Sociology,” “Frontiers in Communication,” “Frontiers in Digital Health,” and “Frontiers in Public Health.” The presented collection includes nine articles by 42 authors from China, Greece, Hungary, Indonesia, Israel, Italy, the Netherlands, Norway, and the United States. Four types of articles are included: six original research articles (Huang et al.; Jiao et al.; Li and Li; Lyu; Standaar; Trenggono et al.), one brief research report (Shraga et al.), one review (Lippai et al.), and one opinion (Galioto et al.). The call for papers was open and not limited to conference participants. As a result, the collection includes studies focused on cases from European countries such as Italy, the Netherlands, and Ukraine, as well as thematically related research from China and Indonesia. The studies are organized according to three themes.

## Theme I: Co-creating cities' social inclusion

In the first study included in this Research Topic, Lippai et al. propose a meta-theory perceiving wellbeing as a socially constructed representation tied to individual and collective choices affecting quality of life and arguing that current public health approaches are insufficient. This study advocates for a new “public wellbeing system” built on co-production, aligning with theories emphasizing user participation to meet societal needs. The model applies to co-creating inclusive cities by addressing collective wellbeing and social positioning via participatory service design.

The next two studies focus on cases from China. Li and Li examined Chinese childcare policies framed by social constructionism, which reveal a system dominated by government and institutional actors, highlighting challenges of urban-rural disparities and unequal resource distribution pertinent to social inclusion. The findings indicate insufficient co-creation involving communities and families in developing and implementing health-oriented childcare despite the acknowledged need for collaboration among diverse actors. Enhancing urban social inclusion requires improving negotiation among all stakeholders and sharing responsibilities.

In the next paper, Lyu shows that access to public health services significantly enhances migrant workers' intention to settle in Chinese cities, fostering urban social inclusion by improving their satisfaction and sense of belonging. While confirming service provision's positive impact, the findings highlight the need to tailor services to migrant workers' needs. Applying co-creation by involving migrant workers in service design/adaptation could be crucial for promoting their urban integration.

## Theme II: Co-creating responses to manage the future workforce

Huang et al.'s study on hospital operational efficiency in Western China identified declining efficiency and suboptimal resource utilization, implicitly impacting the healthcare workforce environment and suggesting a need for strategic shifts in hospital management. The analysis points to factors such as personnel expenses and resource allocation as areas for improvement. Addressing future workforce challenges could thus involve co-creation and engaging professionals in designing quality-focused work systems and resource management.

The study by Trenggono et al. examined how a university rector utilizes communication patterns, including symbols and rituals, as adaptive strategies to manage the academic workforce and preserve institutional culture amidst challenges such as performance decline and scandals. However, the analysis highlights top-down communication efforts, contrasting with co-creation principles. Co-creation theories suggest that managing the future workforce requires moving beyond unilateral communication toward participatory processes, engaging staff in shaping cultural responses, building trust, and defining resilience.

## Theme III: Co-creating digital solutions for social inclusion

The papers included in this section start with Standaar et al., who focus on digital health skills training in Dutch public libraries as a solution to foster social inclusion, revealing that despite identifying diverse, vulnerable groups, these programs struggle to reach beyond older adults due to accessibility issues and client barriers. The findings strongly advocate collaborations (libraries, healthcare, welfare, and community organizations) to enhance reach/diversity, reflecting social policy approaches emphasizing multi-stakeholder co-creation. Effective co-creation thus requires interorganizational partnerships and potentially deeper community engagement.

Jiao et al. provide an analysis of participation drivers in a web-based time bank in China, identifying this digital platform as an explicit form of co-creation aimed at fulfilling unmet social needs and potentially enhancing community social inclusion. Upon analyzing service request narratives, the research reveals that engagement is motivated more by extrinsic rewards (time credits) and intrinsic cues (social connection, personal value) than pure altruism. Understanding these motivations through sociological and social policy lenses is therefore crucial for effectively co-creating digital solutions for social inclusion.

Shraga et al. studied an international, phone-based psychological first aid program for Ukrainian civilians, presenting a digital solution delivered by an informal volunteer group to promote mental wellbeing and social inclusion for a vulnerable population lacking access to formal support. The intervention demonstrates feasibility and positive outcomes, but its informal nature shows limitations of co-creation between volunteers, recipients, and formal systems.

In the final paper, Galioto et al. argue that universities should integrate digital solutions such as social media and innovative technologies into their communication strategies to boost student engagement and sense of belonging, thereby fostering social inclusion within higher education environments. While highlighting the role of university management and researchers in implementing these tools, the emphasis on user interaction/empowerment points toward co-creation over top-down communication. Applying co-creation principles involves actively engaging students in designing/deploying these digital platforms to ensure they promote inclusion and empower diverse voices.

## Conclusion

The research results presented in the articles of this collection allow for the formulation of at least five directions for further research. These are: (1) intersectionality in co-creating inclusive support services (Horvath and Carpenter, 2020; Gergen, 2023); (2) ethical frameworks for inclusive digital co-creation (Deserti et al., 2022; Lindberg, 2024); (3) co-designing platforms and building digital literacy for social inclusion (Jarke, 2021; Maciel, 2024; Suoheimo et al., 2025); (4) scaling sustainable co-produced initiatives (Edelmann and Virkar, 2023; van Gestel et al., 2023); and (5) evaluating co-produced social services and comparing them with traditional services (Loeffler and Bovaird, 2021; Nasi et al., 2024; Greve, 2025).
